# Primary cardiac angiosarcoma: A clinical report of 1 case and review of the literature

**DOI:** 10.1097/MD.0000000000041556

**Published:** 2025-02-14

**Authors:** Jiubo Sun, Jing Li, Xiaofei Wang, Xiaoguang Huo, Wenzhe Xu, Fei Li

**Affiliations:** a No. 2 Department of Oncology, Zibo Central Hospital, Zibo, China; b Department of Traditional Chinese Medicine, Zibo Central Hospital, Zibo, China; c Department of Ultrasound, Zibo Central Hospital, Zibo, China; d No. 1 Department of Cardiology, Zibo Hospital of Shandong Healthcare Group, Zibo, China.

**Keywords:** cardiac angiosarcoma, chemotherapy, computed tomography, magnetic resonance imaging, surgery, ultrasonography

## Abstract

**Rationale:**

Primary cardiac angiosarcoma is very rare with a poor prognosis, and there is no recognized guideline for its diagnosis and treatment. Currently, there is no standardized adjuvant chemotherapy regimen following radical surgery for cardiac angiosarcoma. To date, the literature lacks reports on the use of albumin-bound paclitaxel as an adjuvant chemotherapy agent in this context. This case report aims to document and evaluate the imaging characteristics of the disease and the efficacy of albumin-bound paclitaxel as adjuvant chemotherapy, thereby providing valuable insights for clinical diagnosis and treatment.

**Patient concerns:**

We report a 70-year-old patient with right atrial angiosarcoma, who presented with atrial fibrillation as the initial symptom.

**Diagnoses:**

Transesophageal echocardiography, chest computed tomography, and cardiac magnetic resonance imaging showed a right atrial mass.

**Interventions:**

After radical resection, the patient was given 4 cycles of albumin-bound paclitaxel adjuvant chemotherapy.

**Outcomes:**

Multiple metastases occurred in a short period of time, and the patient died 13 months after surgery. Surgical resection is the most important treatment for cardiac angiosarcoma, and the optimal adjuvant therapy needs to be further studied.

**Lessons:**

The limitation of this case report is its reliance on a single case, resulting in a limited sample size. To comprehensively characterize cardiac angiosarcoma and evaluate the efficacy of albumin-bound paclitaxel chemotherapy, future studies should collect additional cases and conduct long-term follow-up.

## 1. Introduction

The incidence of primary cardiac malignancy is extremely low, with primary cardiac angiosarcoma being 1 of the most prevalent subtypes. The clinical manifestations exhibit a lack of specificity. The prognosis for angiosarcoma is bleak due to its aggressive nature, with a median overall survival ranging from 6 to 14 months.^[1]^ The literature lacks sufficient reports on the use of albumin-bound paclitaxel adjuvant chemotherapy following radical surgery.

## 2. Case report

**Figure 1.**
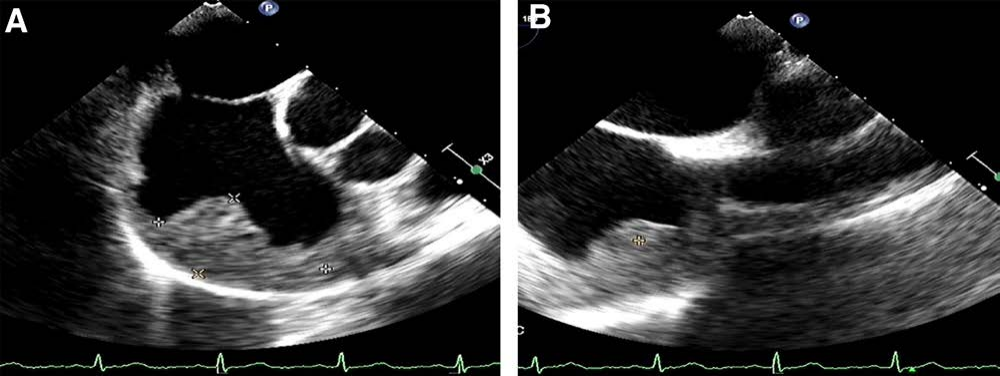
Transesophageal echocardiography of the heart. (A and B) A hypoechoic mass was detected in the right atrium near the opening of the superior vena cava, about 43 × 26 mm in size, with clear boundary, irregular shape, and heterogeneous internal echo.

**Figure 2.**
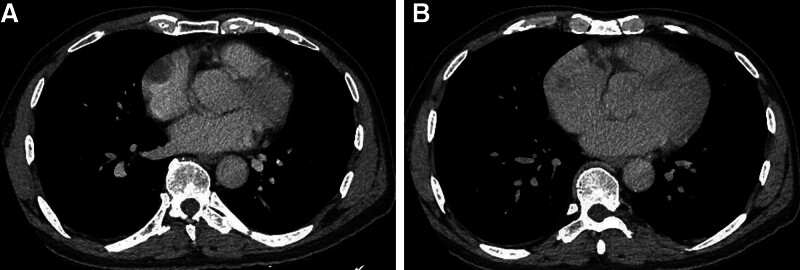
Enhanced CT scan of the chest. (A) After the injection of contrast agent, the right atrium showed a low-density filling defect with an unclear boundary and a lower CT value than the surrounding atrial cavity. (B) The lesion was markedly heterogeneous enhanced on delayed scan, and the maximum cross section was about 3.9 × 2.4 cm. CT = computer tomography.

**Figure 3.**
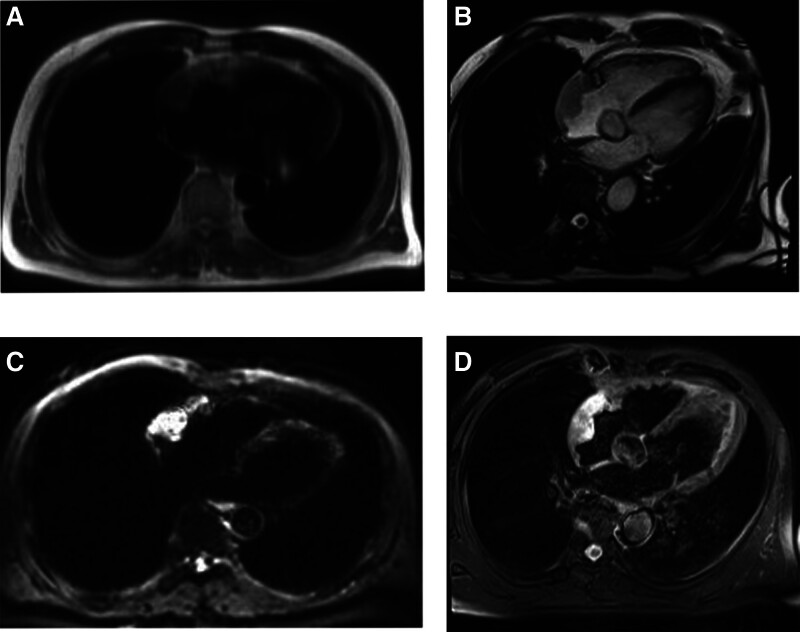
Cardiac MRI examination of the 4-chamber cardiac position. (A) T1WI showed isointensity of the right atrial lesion. (B) The right atrial lesion showed slightly hyperintensity on T2WI. (C) DWI showed high signal intensity in the right atrial lesion. (D) The lesions were significantly enhanced on T1WI + C. The dimensions measured approximately 5.6 × 2.4 cm. MRI = magnetic resonance imaging.

**Figure 4.**
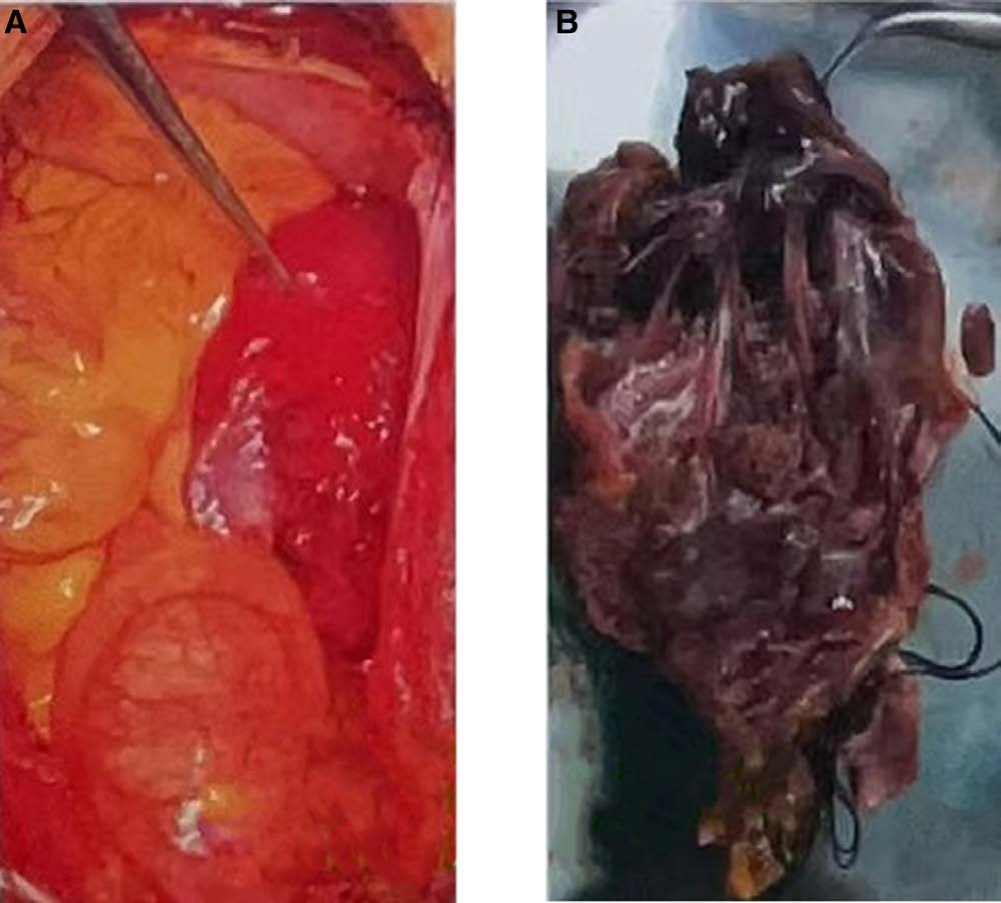
Intraoperative picture. (A) The tweezers indicate the tumor, which has invaded almost the entire right atrium, and the tumor is close to the right atrioventricular groove. (B) Medial view of tumor.

The Ethics Committee of our hospital granted approval for this study (ethics no. 2023 research no. 126). A 70-year-old male patient presented to the hospital on February 17, 2022, with a complaint of “paroxysmal chest tightness persisting for 1 year, exacerbated over the past 3 days.” The electrocardiogram (ECG) revealed atrial fibrillation, which subsequently converted to sinus rhythm following amiodarone administration. The transesophageal echocardiography further revealed a low echo mass in the right atrium, precisely located at the right atrial roof near the opening of the superior vena cava. The mass exhibited a wide base and an approximate oval shape, leading to considerations of thrombus or space-occupying lesion (Fig. 1). Enhanced computed tomography (CT) of the chest revealed an ill-defined, low-density filling defect in the right atrium with indistinct margins. The maximum cross-sectional area measured approximately 3.9 × 2.4 cm. The CT value following contrast agent administration exhibited a lower intensity compared to the surrounding atrial cavity, and the lesion demonstrated evident heterogeneous enhancement on delayed scan, indicative of a space-occupying lesion (Fig. 2). Further improvement of cardiac magnetic resonance imaging (MRI) revealed irregular thickening of the lateral wall of the right atrium, along with patchy abnormal signal lesions exhibiting indistinct boundaries with normal myocardium. The dimensions measured approximately 5.6 × 2.4 cm, and the demarcation was not well-defined. T1-weighted imaging demonstrated isointensity, T2-weighted imaging exhibited slightly elevated signal intensity, and diffusion-weighted imaging displayed high signal intensity. Additionally, there was a reduction in the size of the right atrial cavity, and delayed scans indicated significant enhancement changes within the lesion (Fig. 3), suggestive of a right atrial neoplasm. Following exclusion of distant metastasis and contraindications, surgical intervention involving resection of the right atrial mass + reconstruction + temporary epicardial pacing electrode implantation was performed on March 1, 2022. Intraoperatively, it was observed that a bright red mass with a smooth surface occupied most of the right atrium; its left margin abutted against the right atrioventricular groove while its upper edge extended towards the left atrium. The distance between its right margin and tricuspid valve annulus measured approximately 1 cm whereas its lower border lay roughly 2 cm away from the opening of inferior vena cava. Careful examination of the tumor revealed no invasion into the right coronary artery and tricuspid annulus (Fig. 4).

The patient underwent a successful operation and was subsequently transferred to the intensive care unit (ICU) post-surgery. In the ICU, the patient received ventilator-assisted ventilation to maintain circulatory stability and control heart rate and blood pressure. On the first postoperative day, the patient was successfully extubated and transitioned to oxygen inhalation via bilateral nasal cannulas. The patient was transferred to the general cardiac surgery ward on March 3, 2022. The pericardial and mediastinal drainage tubes were removed on March 5, 2022. Postoperative ECG review confirmed satisfactory sinus rhythm, after which the temporary pacemaker was removed. The patient was discharged on March 7, 2022, with no recurrence of chest pain or atrial fibrillation.

**Figure 5.**
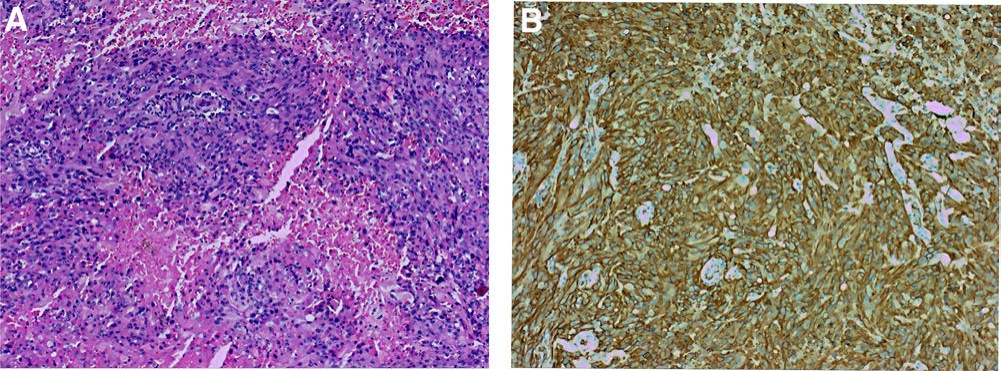
Pathology and immunohistochemistry. (A) Postoperative pathological angiosarcoma of right atrial mass (HE × 100 times). (B) Tumor tissue immunohistochemical CD31 positive (×100 times).

Postoperative pathological analysis demonstrated 3 pieces of gray-pink irregular tissue, measuring a total size of 8.5 × 6.5 × 1.5 cm. The postoperative pathology confirmed angiosarcoma in the right atrium. Immunohistochemical findings exhibited positive staining for CD31, ERG, focal EMA, Vimentin, CD34 vessels; negative staining for CKAE1/AE3, CK8/18, CK5/6; and a Ki-67 proliferation index of 65% (Fig. 5). Four cycles of albumin-bound paclitaxel at a dose of 200 mg on days 1, 8, and 15 every 28 days were administered on June 21, July 19, August 16, and September13 after surgery. In late December 2022, liver metastasis, lung metastasis, and bone metastasis were detected. The patient declined further antitumor treatment and succumbed to the disease on March 30, 2023.

## 3. Discussion

The incidence of primary cardiac tumors is extremely low, ranging from 0.02% to 0.056%, as reported in autopsy studies.^[2,3]^ Approximately 10% of these tumors are malignant.^[4,5]^ Primary malignant cardiac tumors with a prevalence of about 0.01% among all cancer histotypes.^[6]^ Among the different pathological types, cardiac angiosarcoma is the most prevalent (41%), followed by undifferentiated sarcoma (23%) and fibrosarcoma (18%).^[7]^ Notably, the right atrium is the most common site for cardiac angiosarcoma occurrence, observed in 70% to 100% of patients.^[7–9]^

The predominant clinical manifestations encompassed dyspnea and chest pain.^[9]^ With the progression of angiosarcoma, right-sided heart failure frequently ensued. Involvement of the sinus node and atrioventricular conduction system may precipitate malignant arrhythmias.^[7]^ While metastasis can give rise to multi-organ symptoms.

Due to the nonspecific clinical symptoms and signs of primary cardiac angiosarcoma, its detection primarily relies on imaging examinations such as echocardiography, CT, and MRI. Transthoracic echocardiography demonstrated a reported sensitivity of 93%, while transesophageal echocardiography exhibited a sensitivity of 97%.^[10]^ Cardiac angiosarcoma displayed moderate to low echogenicity with a fixed morphology and no discernible activity on echocardiography. The tumor exhibits a broad attachment surface without a peduncle, enabling infiltration into the local myocardium. Previous investigations have demonstrated that cardiac angiosarcoma is characterized by abundant neovascularization and varying degrees of hemorrhage, necrosis, and fibrosis.^[11,12]^ CT scans reveal an isodense or low-density mass in the right atrium, predominantly exhibiting heterogeneous enhancement with flocculent reticular vascular shadowing.^[11,13]^ MRI demonstrates isointensity on T1-weighted images and hyperintensity or slight hyperintensity on T2-weighted images, along with mild-to-moderate heterogeneous enhancement during delayed enhancement.^[12–14]^ The imaging findings of the patient were consistent with the reports. MRI enables clear visualization of tumor size, shape, location, and extent, as well as its relationship with the myocardium, pericardium, and adjacent major vessels. When combined with comprehensive cardiac imaging techniques such as 1-stop imaging, cardiac function analysis, myocardial perfusion assessment, and myocardial activity examination can be performed. Notably in 2004, both the European Society of Cardiology and the Cardiovascular Magnetic Resonance Society designated MRI as the primary modality for evaluating cardiac tumors.^[15]^

Due to the rarity of primary cardiac angiosarcoma, current studies mainly consist of retrospective studies and case reports, with no standardized treatment method established in clinical practice. Comprehensive treatment approaches are preferred in most research reports, which may include surgery, chemotherapy, targeted therapy, immunotherapy, and radiotherapy.^[16]^ Radical surgery is considered the most important treatment if there is no obvious metastasis or surgical contraindication.^[17]^ Hasan et al reported on 26 patients with primary cardiac sarcoma who underwent 31 curative-intent surgical procedures. Among the cohort, 7 patients had previously undergone resection or biopsy at an external institution. Subsequent surgical interventions included secondary resection in 10 patients, triple resection in 2 patients, and quadruple resection in 1 patient, all without any operative mortality. Additionally, 6 patients who were deemed inoperable underwent biopsy and palliative tumor debulking, followed by definitive chemoradiotherapy. The median survival was observed to be 3 years, with estimated survival rates of 90%, 73%, 31%, and 17% at intervals of 6 months and at the end of years 1, 5, and ten respectively.^[18]^ In a study by Hendriksen et al, it was demonstrated that surgical patients exhibited a median survival time of 16 months with a corresponding 5-year survival rate of 13.3%, which surpassed the figures for nonsurgical patients (11 months and 2.4%); The median survival time of patients receiving adjuvant therapy following surgery was 19 months, demonstrating a significant improvement compared to those without adjuvant therapy (8 months), However, there was no statistically significant difference observed in the 5-year survival rate.^[19]^

Currently, there is no standardized chemotherapy regimen for angiosarcoma, and available clinical options include taxanes, platinums, doxorubicin, gemcitabine, among others. Ishibashi Naoya et al reported a case of locally advanced primary cardiac angiosarcoma that was inoperable due to its location. After receiving 5 cycles of docetaxel, echocardiography and CT scans showed a significant reduction in tumor size, indicating a partial response. Chemotherapy was temporarily discontinued for 8 months because of docetaxel-induced neutropenia and general fatigue. Subsequently, docetaxel treatment was resumed and continued for an additional 4 cycles; however, the tumor developed resistance to docetaxel, and liver metastases were observed.^[20]^ Suzuki et al reported 3 cases of cardiac angiosarcoma where patients were admitted directly to the ICU due to hemodynamic instability leading to circulatory failure. These patients received weekly paclitaxel therapy, which resulted in clinical improvement and partial remission, as evidenced by imaging studies.^[21]^ Wang et al reported a case of primary cardiac angiosarcoma with multiple metastases. The patient was administered paclitaxel for injection at a dose of 90 mg/m² on days 1, 8, and 15 of a 28-day cycle. Despite 6 months of treatment, disease progression was observed. Subsequently, second-line therapy comprising vinorelbine and bevacizumab was initiated; however, this regimen also resulted in an unsatisfactory response. Ultimately, the patient succumbed to respiratory failure, with an overall survival time of 7 months^[22]^ (Table 1).

**Table 1:** Cases of unresectable cardiac angiosarcoma reported.

First author, year	Number	Location	Treatment	Outcome (mo)	References
Ishibashi, 2007	1	Right atrium	Docetaxel 30 mg/m^2^ d1, d8, d15/4 wk (line 1), docetaxel 30 mg/m^2^ d1, d8, d15/4 wk (line 2), paclitaxel, irinotecan, interleukin-2 (line 3)	PFS 8	[20]
				OS:31	
Suzuki, 2021	1	Right atrium	Paclitaxel 100 mg/m^2^ d1, d8, d15, 22, 29, d36/7 wk	PR	[21]
				OS:14.3	
	1	Right atrium	Paclitaxel 100 mg/m^2^ d1, d8, d15, d22, d29, d36/7 wk	PR	
				OS:7.5	
	1	Right atrium	Paclitaxel 100 mg/m^2^ d1, d8, d15, d22, d29, d36/7 wk	PR	
Wang, 2017	1	Right atrium	Paclitaxel 90mg/m^2^ d1, d8, d15/4 wk (line 1), vinorelbine 25 mg/m^2^ d1, d8/3 wk and bevacizumab 10 mg/kg d1/3 wk (line 2)	PFS:6	[22]
				OS:7	

OS = overall survival, PFS = progression-free survival, PR = partial response.

After undergoing radical surgery, the patient received adjuvant chemotherapy with single-agent injectable paclitaxel (albumin-bound), which was well tolerated. No adverse reactions such as nausea, vomiting, myelosuppression, or peripheral neurotoxicity were observed during the chemotherapy. However, despite the absence of these side effects, multiple metastases were detected in the patient by the end of December 2022, only 3 months after completing chemotherapy at the end of September 2022. The progression-free survival lasted for 9 months, while the overall survival was limited to only 13 months. Through this case report, we aim to enhance clinicians’ understanding of cardiac angiosarcoma and provide valuable insights for the selection of postoperative chemotherapy regimens.

Primary cardiac angiosarcoma is an exceedingly rare malignancy with a dismal prognosis. Currently, there is no universally accepted guideline for its diagnosis and treatment. Further clinical studies are warranted to ascertain the optimal treatment plan and modality.

## Author contributions

**Conceptualization:** Jing Li.

**Data curation:** Jing Li.

**Investigation:** Xiaofei Wang.

**Methodology:** Fei Li.

**Resources:** Wenzhe Xu.

**Writing – original draft:** Jiubo Sun.

**Writing – review & editing:** Xiaoguang Huo, Fei Li.

## References

[1] Timóteo AT Branco LM Bravio I. Primary angiosarcoma of the pericardium: case report and review of the literature. Kardiol Pol. 2010;68:802–5. 20648441

[2] Reynen K. Frequency of primary tumors of the heart. Am J Cardiol. 1996;77:107. 8540447 10.1016/s0002-9149(97)89149-7

[3] Lam KY Dickens P Chan AC. Tumors of the heart. A 20-year experience with a review of 12,485 consecutive autopsies. Arch Pathol Lab Med. 1993;117:1027–31. 8215825

[4] Rahouma M Arisha MJ Elmously A. Cardiac tumors prevalence and mortality: a systematic review and meta-analysis. Int J Surg. 2020;76:178–89. 32169566 10.1016/j.ijsu.2020.02.039

[5] Heaton JN Dhaduk N Okoh AK. Characteristics, management, and outcomes among admissions for primary cardiac tumors: results from the national inpatient sample. J Card Surg. 2021;36:3586–92. 34314042 10.1111/jocs.15862

[6] Scicchitano P Sergi MC Cameli M. Primary soft tissue sarcoma of the heart: an emerging chapter in cardio-oncology. Biomedicines. 2021;9:774. 34356838 10.3390/biomedicines9070774 PMC8301302

[7] Garcia Brás P Branco LM Galrinho A. Malignant primary and metastatic cardiac tumors: a single-center 27-year case review. Oncology (Huntingt). 2023;101:292–302. 10.1159/000528915 36657399

[8] Rodriguez Ziccardi M Tariq MA Limaiem F Ahmed SW. Cardiac cancer. 2023 Jan 1. In: StatPearls. StatPearls Publishing; 2024. 30725829

[9] Chambergo-Michilot D De la Cruz-Ku G Sterner RM Brañez-Condorena A Guerra-Canchari P Stulak J. Clinical characteristics, management, and outcomes of patients with primary cardiac angiosarcoma: a systematic review. J Cardiovasc Thorac Res. 2023;15:1–8. 37342661 10.34172/jcvtr.2023.30531 PMC10278191

[10] Meng Q Lai H Lima J Tong W Qian Y Lai S. Echocardiographic and pathologic characteristics of primary cardiac tumors: a study of 149 cases. Int J Cardiol. 2002;84:69–75. 12104067 10.1016/s0167-5273(02)00136-5

[11] Yu JF Cui H Ji GM. Clinical and imaging manifestations of primary cardiac angiosarcoma. BMC Med Imaging. 2019;19:16. 30764784 10.1186/s12880-019-0318-4 PMC6375141

[12] O’Donnell DH Abbara S Chaithiraphan V. Cardiac tumors: optimal cardiac MR sequences and spectrum of imaging appearances. AJR Am J Roentgenol. 2009;193:377–87. 19620434 10.2214/AJR.08.1895

[13] Hoey ET Mankad K Puppala S Gopalan D Sivananthan MU. MRI and CT appearances of cardiac tumours in adults. Clin Radiol. 2009;64:1214–30. 19913133 10.1016/j.crad.2009.09.002

[14] Li X Chen Y Liu J. Cardiac magnetic resonance imaging of primary cardiac tumors. Quant Imaging Med Surg. 2020;10:294–313. 31956550 10.21037/qims.2019.11.13 PMC6960432

[15] Pennell DJ Sechtem UP Higgins CB; Society for Cardiovascular Magnetic Resonance; Working Group on Cardiovascular Magnetic Resonance of the European Society of Cardiology. Clinical indications for cardiovascular magnetic resonance (CMR): consensus panel report. Eur Heart J. 2004;25:1940–65. 15522474 10.1016/j.ehj.2004.06.040

[16] Mayer F Aebert H Rudert M. Primary malignant sarcomas of the heart and great vessels in adult patients: a single-center experience. Oncologist. 2007;12:1134–42. 17914083 10.1634/theoncologist.12-9-1134

[17] Ryu SW Jeon BB Kim HJ. Surgical outcomes of malignant primary cardiac tumor: a 20-year study at a single center. Korean J Thorac Cardiovasc Surg. 2020;53:361–7. 33115972 10.5090/kjtcs.20.061 PMC7721522

[18] Hasan SM Witten J Collier P. Outcomes after resection of primary cardiac sarcoma. JTCVS Open. 2021;8:384–90. 36004100 10.1016/j.xjon.2021.08.038 PMC9390277

[19] Hendriksen BS Stahl KA Hollenbeak CS. Postoperative chemotherapy and radiation improve survival following cardiac sarcoma resection. J Thorac Cardiovasc Surg. 2021;161:110–9.e4. 31928808 10.1016/j.jtcvs.2019.10.016

[20] Ishibashi N Mitachi Y Sugawara S. A case of cardiac angiosarcoma successfully treated with docetaxel. Gan To Kagaku Ryoho. 2007;34:1849–52. 18030022

[21] Suzuki T Yamamoto Y Sakamoto N. Dramatic recovery from cardiovascular collapse: paclitaxel as an urgent treatment for primary cardiac angiosarcoma. Intern Med. 2021;60:67–71. 32830183 10.2169/internalmedicine.5420-20 PMC7835460

[22] Wang C Shi M Yang C. Comprehensive treatment of unresectable cardiac angiosarcoma: a case report and review of literature. Mol Clin Oncol. 2017;7:859–63. 29181180 10.3892/mco.2017.1390 PMC5700288

